# Exploring Stroke Risk through Mendelian Randomization: A Comprehensive Study Integrating Genetics and Metabolic Traits in the Korean Population

**DOI:** 10.3390/biomedicines12061311

**Published:** 2024-06-13

**Authors:** Hyo-Jeong Ban, Siwoo Lee, Hee-Jeong Jin

**Affiliations:** Korean Medicine (KM) Data Division, Korea Institute of Oriental Medicine, Daejeon 34054, Republic of Korea; hjban@kiom.re.kr (H.-J.B.); bfree@kiom.re.kr (S.L.)

**Keywords:** cardioembolic stroke, constitutional type, convolutional neural network analysis, Mendelian randomization, cardiometabolic condition

## Abstract

Numerous risk factors play a role in the causation of stroke, and the cardiometabolic condition is a one of the most important. In Korea, various treatment methods are employed based on the constitutional type, which is known to differ significantly in cardiometabolic disease. In this study, we compared the estimates obtained for different groups by applying the Mendelian randomization method to investigate the causal effects of genetic characteristics on stroke, according to constitutional type. In clinical analysis, the subtypes differ significantly in diabetes or dyslipidemia. The genetic association estimates for the stroke subtype risk were obtained from MEGASTROKE, the International Stroke Genetics Consortium (ISGC), UKbiobank, and BioBank Japan (BBJ), using group-related SNPs as instrumental variables. The TE subtypes with higher risk of metabolic disease were associated with increased risk (beta = 4.190; s.e. = 1.807; *p* = 0.035) of cardioembolic stroke (CES), and the SE subtypes were associated with decreased risk (beta = −9.336, s.e. = 1.753; *p* = 3.87 × 10^−5^) of CES. The findings highlight the importance of personalized medicine in assessing disease risk based on an individual’s constitutional type.

## 1. Introduction

Stroke is the second leading cause of death worldwide, and it is responsible for approximately 11% of deaths [1]. In Korea, stroke is the second highest cause of death, and the total number of stroke events continues to rise due to a rapidly aging population [2]. Cardiometabolic conditions, which encompass a cluster of metabolic and cardiovascular abnormalities, such as abdominal obesity, insulin resistance, hypertension, dyslipidemia, and atherosclerosis [3,4], are significant risk factors for stroke [5]. Although there are various subtypes of stroke, including ischemic stroke (IS), cardioembolic stroke (CES), aortic stroke (LAS), and all stroke (AS), and the causes of each subtype are different, the cardiometabolic condition is a major risk factor for overall stroke.

The cardiometabolic condition is considered a potent modifiable risk factor, as stroke development in this case can be prevented; for example, the blood pressure can be lowered via weight loss in people with obesity and via reductions in the systolic and diastolic blood pressure [6,7,8]. Conversely, age, gender and race, ethnicity, and genetic predisposition are non-modifiable (unchangeable) risk factors for stroke [9]; these are known to cause approximately 10% of strokes [8]. Accordingly, various researchers have focused on the management of variable risk factors, since most strokes are attributable to modifiable risk factors [10,11,12,13,14,15,16,17]. 

In Korea, individuals receive personalized treatment and healthcare based on their constitutional type. This diagnosis is dependent upon various aspects related to health, including psychological, social, and physical factors, such as body structure, function, and metabolism. The constitutional type can be inherited, as demonstrated via univariate and multivariate model-fitting analyses in a twin study [18]. Numerous previous clinical studies have reported the constitutional type as a risk factor for cardiometabolic conditions, such as diabetes [19,20,21,22], obesity [23,24], hypertension [25], and cardiovascular disease (CVD) [26,27]. In a cohort study that compared the risk prediction models for metabolic disorders based on the constitutional type, 14-year follow-up data indicated that the predictive accuracy was higher for the Tae-eum (TE) type than for the So-eum (SE) and So-yang (SY) types [28]. From a genome research perspective, some studies have reported associations with constitutional type. In a study by Kim et al., the SNPs involved in neuronal function, cell signaling function, and the development process of each constitutional type were found to be, TE, SE, and SY, respectively [29].

In recent years, the International Stroke Genetics Consortium (ISGC) [30] and the MEGASTROKE Consortium [31], in a large-scale international collaboration, published statistics summarizing the stroke meta-analysis, disclosed the associated loci, and shared the genetic mutations for each stroke subtype. However, it was unable to explain that a combination of cardiometabolic conditions present a causal risk factor for stroke. 

The aim of the present study was to investigate constitutional types and their contribution to the risk of stroke, to help enhance the comprehension of stroke etiology and provide insights for the development of more effective preventive measures. First, the clinical information was analyzed to confirm the distribution of cardiometabolic disease-related traits in constitutional types. Then, the causal relationship between the constitutional type and stroke was investigated using the Mendelian Randomization (MR) method. MR is similar to a randomized controlled trial but minimizes causality and uses genetic variation as a proxy for exposure to study its effect on the outcomes. 

## 2. Materials and Methods

### 2.1. Study Participants

The study participants were recruited as part of the Korean Genome and Epidemiology Study (KoGES) [32], a population-based cohort study spanning the Ansan and Ansung regions; this genome-wide association study (GWAS) was conducted from 2001 to 2019. In total, 5797 participants (2607 men and 2940 women), for whom information on the constitutional types were collected by the Korea Institute of Oriental Medicine (KIOM) in cooperation with the KoGES, were selected. The participants were healthy and had no history of cancer treatment or disease. Three constitutional types, namely TE, SE, and SY, were identified using the Korean Sasang Constitutional Diagnostic Questionnaire (KS-15) [33]. 

The KS-15 is a well-validated, shortened, and cost-effective screening instrument that is used to assess the constitutional type with clinical relevance (Cronbach a = 0.630). It consists of 15 items associated with individuals’ anthropometric awareness of height and weight, 6 questions regarding personality (broad-minded/delicate, act quick/slow, active/passive, extraverted/introverted, masculine/feminine, excitable/rational), and 8 symptom-related questions concerning physiological functions (good digestion, appetite, excessive sweating, feeling when seated, abdominal tension during bowel movements, urination per night during sleep, dislike of cold and heat, preference for temperature when drinking water). The KS-15 derives the scores of each constitutional type, which range from 0 to 1.0 for each person; the sum of scores of the three constitutional types that is derived by the KS-15 for each individual is 1.0 (Table S1).

### 2.2. Clinical Data Analysis

We analyzed cardiometabolic-related clinical information according to the constitutional type of the cohort participants; this information included the Body Mass Index (BMI), Total cholesterol (TC), High-density lipoprotein cholesterol (HDL-C), Low-density lipoprotein cholesterol (LDL-C), Triglyceride (TG), Fasting glucose (FG), systolic blood pressure (SBP), and diastolic blood pressure (DBP). The clinical information associated with these metabolic traits is detailed in Table S2. Using the R package, we performed a Kruskal–Wallis analysis [34] for BMI, the glucose, TG, TC, LDL-C, and HDL-C levels, and blood pressure. Although a substantial number of older individuals aged ≥60 years were present in the cohort, we did not conduct an age-stratified analysis; this was due to the lack of statistically significant differences in the average age of individuals among the constitutional types. To calculate the odds ratios of hypertension, dyslipidemia, and impaired fasting glucose (IFG), we used the Genodds Library [35] in the R package (version 4.1.3). Hypertension was defined as SBP > 140 mm Hg or DBP > 90 mm Hg, and dyslipidemia was defined as TC > 200 mg/dL, LDL-C > 130 mg/dL, HDL-C < 60 mg/dL, and TG > 150 mg/dL. IFG was defined as fasting glucose levels > 100 mg/dL [36]. 

### 2.3. GWAS and Experimental Validation

An association analysis was conducted using linear regression modeling to identify the single-nucleotide polymorphisms (SNPs) associated with the KS-15 scores, adjusting for age, sex, and the recruitment area using PLINK 1.07 [37] software. After organizing the dataset of significant SNPs (*p* < 0.0001), we created a feature set to understand how the functional area information provided by the convolutional neural network GWAS (cnnGWAS) program could be used. The cnnGWAS model [38], a hierarchical pattern detector capable of the learning patterns common to disease-related locations, was employed to detect functional patterns within the SNP block for each constitutional type. The acquired functional scores were considered as evidence of a closer association with the functional biological patterns within a constitution displaying statistical associations among gene regions in the GWAS (Table S3).

To address the absence of a replicated cohort study, whole-genome sequencing was conducted to validate the selected SNP region in individuals who underwent constitutional diagnosis. DNA samples from 30 SE type individuals, 60 TE type individuals, and 30 SY type individuals were obtained from the Korean Medicine Data Center [39]. The Korean Medicine Data Center houses diverse clinical and blood parameter data that have been compiled from a consortium comprising medical centers and community-based cohorts in Korea. Sequencing was performed using the HiSeq X Ten platform (Illumina, San Diego, CA, USA). Mapping and variant calling, including alignment, duplicate marking, and baseline quality recalibration, were executed using the GATK [40] pipeline (version 4.2.4.1). Subsequently, we confirmed the presence of statistically significant and functionally confirmed SNPs in individuals diagnosed with the actual constitutional type. SNPnexus [41] is a web-based variant annotation tool that collects datasets from various sources and allows users to annotate and evaluate them. This annotation tool provides not only functional change scores in the coding region, but also non-coding scoring analysis results, such as fitCons [42], GWAVA [43], CADD [44], and DeepSEA [45]. The functional variants obtained through the deep learning training program cnnGWAS for different phenotypes were further validated for their functionality in non-coding regions using the SNPnexus tool.

### 2.4. Genetic Instrument Variables

We conducted a GWAS (*p* < 0.0001) using the KS-15 values from the KoGES, selecting SNPs that reflect phenotypic characteristics based on the functional scores (score > 0.5) obtained through cnnGWAS. SNPs with variations (AF > 0.05) in the sequencing data of individuals whose constitutional types were determined at the hospital were designated as instrumental variables (IVs). Details of the IVs are provided in Table S4.

### 2.5. Outcomes

IEU OpenGWAS (https://gwas.mrcieu.ac.uk/ (accessed on 26 July 2023)) is a curated open-source repository of GWAS summary statistics managed by the University of Bristol. It contains data pertaining to ischemic stroke (IS; id: ebi-a-GCST005843, ebi-a-GCST006908, ebi-a-GCST90018644), cardioembolic stroke (CES; id: ieu-a-1109), and stroke (any stroke; id: ebi-a-GCST006906, ebi-a-GCST90038613). The GWAS summary data for BioBank Japan (BBJ) [46], MEGASTROKE [31], and International Stroke Genetics Consortium (ISGC) [30] were obtained from OpenGWAS. Linkage disequilibrium proxy SNPs were used when a specific SNP was not present in a dataset, with the criteria set as r2 > 0.6 and the minor allele frequency (MAF) > 0.01. To harmonize the exposure and resulting SNP effects, incorrect effect allele types were removed, and palindromic SNPs were considered.

### 2.6. MR and Sensitivity Analyses

Our MR study design utilized beta estimates for specific IVs of exposure. The present study was performed in accordance with the Strengthening the Reporting of Observational Studies in Epidemiology using MR (STROBE-MR) guidelines (Table S7 STROBE Checklist). We conducted a two-sample MR analysis using the GWAS summary dataset (Table S4) for the outcomes, along with their corresponding beta estimates and standard errors (s.e.). To estimate the causal relationship while reflecting the strength of the genetic instrumental variables, we calculated the F-statistic [47]; this indicates the relative degree of bias, using the MR library of the R package. The analysis was performed using the MRbase [48] application, which includes several sensitivity analyses that assess horizontal pleiotropy and other assumption violations. We initially conducted the simplest inverse variance weighting (IVW) analysis to obtain MR estimates. Fixed-effects IVW assumes that each SNP provides the same estimate. However, this method does not account for horizontal pleiotropy (or other violations of assumptions). We additionally explored the MR Egger [49] method and the median-based estimator [50]. The MR Egger method enables the horizontal pleiotropy to be determined and presents a causal effect that is unbiased by imbalances or directional effects across all SNPs. Moreover, the median-based estimator ensures the unbiased estimation of the causal effect.

## 3. Results

### 3.1. Clinical Characteristics of Cardiometabolic Disease among the Constitutional Types

**Figure 1:**
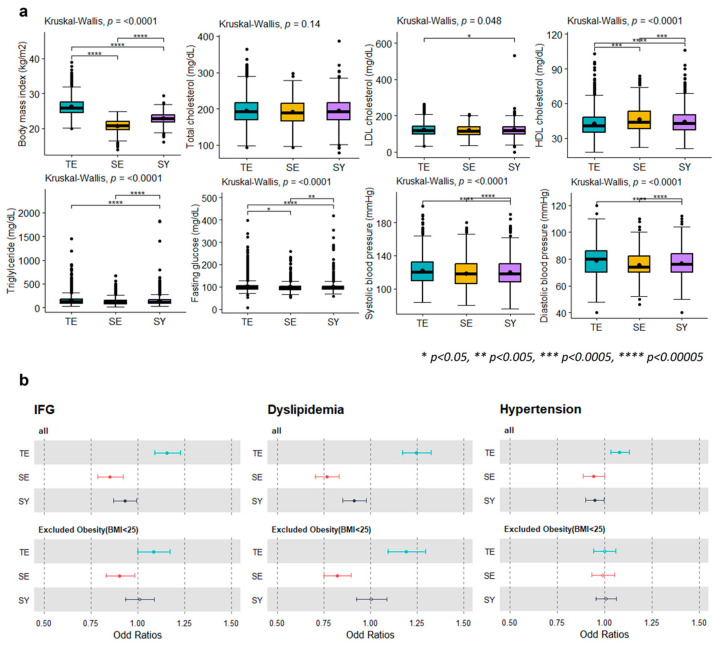
Clinical characteristics of cardiometabolic traits among constitutional types. (**a**) The distributions of cardiometabolic characteristics (BMI; total-C, LDL-C, HDL-C, TG, fasting glucose levels; SBP; and DBP) among the constitutional types show significant differences (Kruskal–Wallis test, *p* < 0.05). (**b**) Forest plots depicting the odd ratios show differences in disease risk (hypertension and dyslipidemia and IFG) among the subtypes. A significant difference (closed point; *p* < 0.05) was observed even when individuals with obesity were excluded (BMI < 25 kg/m^2^), considering that BMI is a risk factor for cardiometabolic diseases.

Prior to assessing disease risk, we confirmed the distribution of cardiometabolic disease-related traits (BMI; TC, HDL-C, LDL-C, TG, and fasting glucose levels; SBP; and DBP) according to the constitutional type. Significant differences in BMI (*p* < 2.2 × 10^−16^), LDL-C (*p* = 0.048), HDL-C (*p* = 4.78 × 10^−14^), TG (*p* = 1.52 × 10^−13^), FG (3.06 × 10^−9^), SBP (*p* = 7.86 × 10^−8^), and DBP (*p* = 6.13 × 10^−16^) were observed (Figure 1a, Table S2), which indicated that each constitutional type poses a distinct risk regarding the cardiometabolic traits.

To verify the actual differences in the disease risk, the distributions of the odds ratio for hypertension (TE type; OR: 1.08, CI: 1.034–1.129, *p* = 0.0006, SE type; OR: 0.941, CI: 0.889–0.995, *p* = 0.034), dyslipidemia (TE type; OR: 1.245, CI: 1.170–1.325, *p* < 0.0001, SE type; OR: 0.768, CI: 0.706–0.835, *p* < 0.0001) and IFG (TE type; OR: 1.158, CI: 1.091–1.229, *p* = 1.37 × 10^−6^, SE type; OR: 0.851, CI: 0.785–0.922, *p* = 7.21 × 10^−5^, SY type; OR: 0.932, CI: 0.872–0.996, *p* = 0.037) were examined, according to the constitutional type (Figure 1b). The results confirmed that the TE type is associated with a high risk of cardiometabolic disease and that the TE type is associated with a low risk of cardiometabolic disease. When obesity (BMI > 25kg/m^2^), a risk factor for cardiometabolic diseases, was excluded, there was a significant change in the prevalence of hypertension. However, the significance of dyslipidemia and IFG in the TE and SE types was maintained. 

### 3.2. Identification of Functional Non-Coding Variants from Constitutional Types

Using cnnGWAS, we identified causal regulatory variants that influenced the biological features of the three constitutional types (TE, SE, and SY). The overall analysis proceeded in the following order: (1) GWAS, (2) feature set construction, (3) deep learning for non-coding variant scoring, (4) functional annotation, and (5) validation (Figure S1). The CNN algorithm, when applied to each constitutional type, resulted in an area under the curve of >0.9 (SE: 98.96%, SY: 98.71%, and TE: 99.65%) for all constitutional types. 

In total, 763 SNPs were identified in the SE type, 455 SNPs were identified in the TE type, and 395 SNPs were identified in the SY type (Figure S2a, Table S3). Notably, associated SNPs that were not detected in the previous GWAS, which solely considered statistical significance (and not SNP functions), were identified in our study. For the SE and TE types, 124 overlapping SNPs were identified, whereas for the TE and SY types, 24 overlapping SNPs were found; however, the SE and SY types did not show any overlapping SNPs (Figure S2a). When genes related to each constitutional type were mapped to those reported in the Genetic Associated Database (GAD) [51], they were mostly distributed in metabolic and cardiometabolic functions (Figure S2b, Table S3). The functional non-coding variants determined according to the constitutional type and learned via cnnGWAS showed high scores in the fitCons, GWASVA, CADD, and DeepSEA results. Due to the absence of a replicated cohort study, whole-genome sequencing was performed to validate the selected SNP regions in individuals diagnosed with constitutional types. Screening for SNPs with variant loci present across all validation participants yielded 26 SNPs associated with the TE constitutional type, 28 SNPs associated with the SE type, and 29 SNPs associated with the SY type (Table S4).

### 3.3. Causal Relationship between Constitutional Type and Stroke Obtained through the MR Analysis

**Figure 2:**
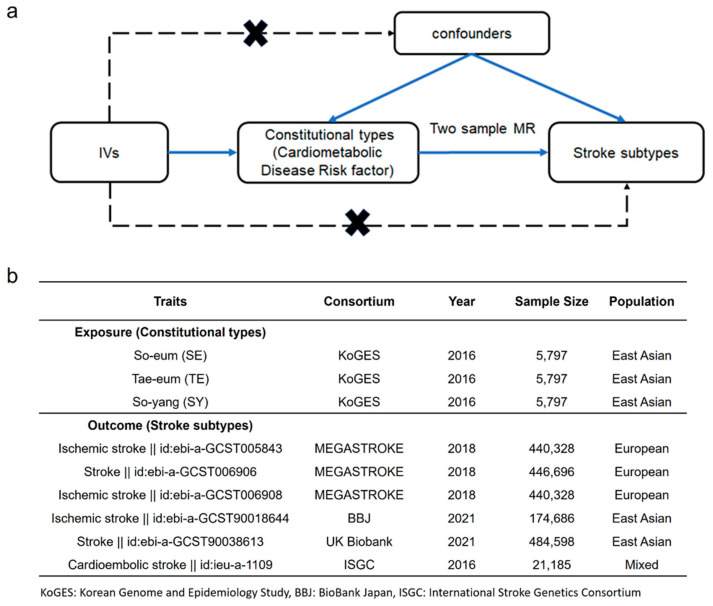
Directed acyclic flowchart of Mendelian Randomization (MR) for the causal relationship between constitutional type and stroke subtype. (**a**) Instrumental variables (IVs) correlated with the constitutional type but not with confounding factors other than those affecting stroke subtype. (**b**) Exposure and outcome datasets were obtained from KoGES and stroke cohorts. (MEGASTROKE, BBJ, Biobank, ISGC).

We tested the hypothesis suggesting that the genetic factors specific to each constitutional type may influence the metabolic traits of blood, consequently leading to variations in stroke risk (Figure 2a). The MR analysis used the beta estimate for each constitutional type as an IV. The GWAS summary data from the stroke cohorts (MEGASTROKE, BBJ, UK Biobank, ISGC) were used for the analysis, which focused on the results of the risk factors for the various stroke subtypes, such as ischemic stroke (IS) and cardioembolic stroke (CES) (Figure 2b). To check the level of instrument bias in the IVs according to the constitutional type, the F-statistic was calculated in the MR analysis. For the IVs of the constitutional type, all had an F-statistics > 13 (Table S6); this allowed us to avoid weak IVs.

**Figure 3:**
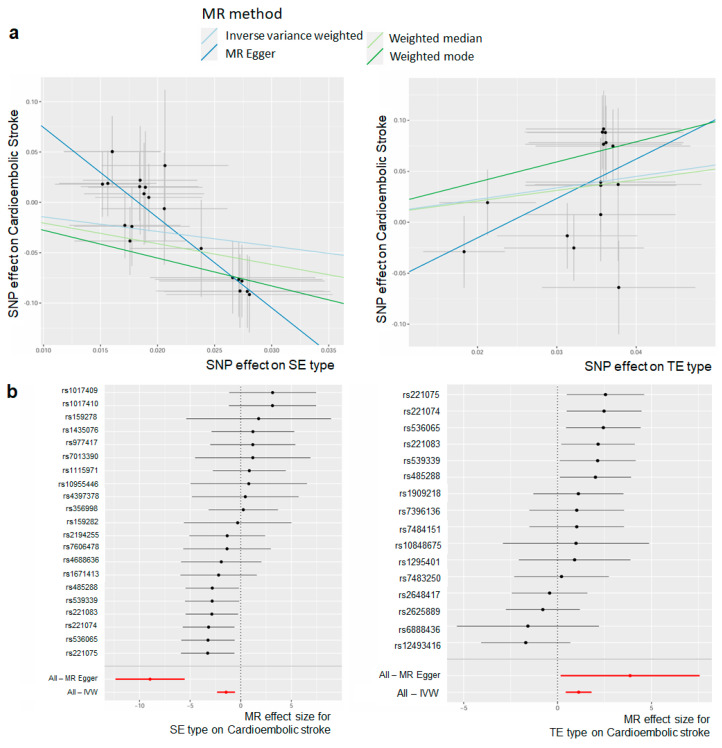
Association between constitutional type and stroke subtype, as determined using two-sample Mendelian Randomization (MR). (**a**) A scatter plot of two sample MR results for the TE type and SE type for CES. The slope of different colored lines represents the estimated MR effect of different MR methods (inverse variance weighted, weighted median, MR Egger, weighted mode). (**b**) Forest plot of MR effect size for constitutional types on cardioembolic stroke. Each point and line indicates the effect (beta) and 95% confidence interval of genetic instruments. Red points and lines show the combined causal estimate and 95% confidence interval range using all SNPs in a single instrument.

The causality estimates differed for each constitutional type, with CES exhibiting a positive correlation with TE type (beta = 4.190; s.e. = 1.807; *p* = 0.035) and SY type (beta = 2.834, s.e. = 1.908; *p* = 0.154), and a negative correlation with SE type (beta = −9.336, s.e. = 1.753; *p* = 3.87 × 10^−5^) (Figure 3a, Table S6). Additionally, no significant causality was observed between the other stroke subtypes and SY type, with a negative relationship observed with SE type (IS; beta = −1.304, s.e. = 0.516; *p* = 0.019). The results suggest that, when a genetic predisposition for the TE type is present, there is a higher risk of CES compared to the other constitutional types. The IVs of each TE type and SE type were separated and are shown in a forest plot (Figure 3b). For TE type, the rs221075 SNP among the IVs had the highest positive effect and, for the SE type, the rs221075 SNP among the IVs had the lowest negative effect. Even for the same SNP, it could be confirmed that the directionality was different depending on the constitutional type. The results of the MR analysis that was performed using four different methods for each stroke subtype according to the constitutional types are presented in Table S6. 

## 4. Discussion

Cardiometabolic disease-related traits are a major risk factor for stroke. In a recent meta-analysis, hypertension, dyslipidemia, diabetes mellitus, and cardiac disease were associated significantly with stroke [8]. Previous clinical studies have revealed that constitutional type is a risk factor for cardiometabolic diseases. Our study aimed to evaluate the potential causal effect of genetically determined constitutional type on the risk of stroke via the MR method.

Firstly, in an analysis of the clinical information, we observed statistically significant differences in risk of diabetes, hypertension, and dyslipidemia between the TE and SE constitutional types. Moreover, significant differences in cardiometabolic traits, such as serum cholesterol, blood glucose, and blood pressure, were observed, according to constitutional type. Cardiometabolic traits, including hypertension [52,53], diabetes [54,55], and dyslipidemia [56,57], are the most important modifiable risk factors for stroke; however, these differed in their contribution depending on the subtype. Moreover, studies have reported that, as the number of cardiometabolic traits increases, the risk and severity of stroke increases [58,59,60]. 

For the selection of IVs in the MR analysis, the variants associated with constitutional type were identified using cnnGWAS. cnnGWAS employs a deep learning approach to identify potential causal non-coding variants and determine their functional activities; it has been used to predict the potential causal variants in major psychiatric disorders and autoimmune diseases [38], including amyotrophic lateral sclerosis (ALS) [61]. The functional non-coding variants that were determined according to constitutional type using cnnGWAS also showed high scores in the fitCons [42], GWASVA [43], CADD [44], and DeepSEA [45] results. These programs can be used to predict the functional effects of non-coding variants based on our functional knowledge of the variants. In addition, the constitutional-type-specific genetic variants were implicated in the regulation of genes associated with cardiometabolic diseases, according to cnnGWAS and GAD results.

By utilizing clinical outcomes that are indicative of the risk associated with various cardiometabolic diseases across constitutional types, we used constitution-specific genetic variants as exposure IVs within the MR analysis. The MR analysis yielded discernible causal estimates for each constitutional type. Notably, within the TE type, both the CES and IS subtypes exhibited statistically significant causal relationships, with CES demonstrating a notably higher magnitude of effect. Conversely, within the SE type, the CES and IS subtypes displayed significant causal associations, albeit with negative causal estimates; this was in contrast to the TE type. Based on the findings, it is plausible that the genetic variants associated with different constitutional types may contribute to the risk of stroke by influencing the regulation of genes involved in cardiometabolic pathways.

Although MR has recently been used to investigate the causal effects that cardiometabolic traits exert on the risk of stroke [62,63,64,65,66], most studies have investigated the causal relationships among individual cardiometabolic traits. Furthermore, such studies have been unable to explain why certain combinations of cardiometabolic traits represent a causal risk factor for stroke. Overall, our findings support the concept of precision medicine, in which approaches to treatment are applied based on constitutional type. Understanding the genetic basis of such constitutional types could aid in the identification of individuals with certain cardiometabolic traits and at a high risk of stroke.

## 5. Limitation

This study acknowledges the absence of a replicated cohort and highlights the importance of conducting further research in order to validate the identified SNPs. Additionally, the present study focused on the Korean population, and generalization to other ethnic groups may require additional investigations. To achieve this, we are collecting and analyze clinical information from other ethnic groups and are working to analyze the genetic information. Furthermore, future studies should investigate the functional significance of the identified SNPs and their involvement in the specific biological pathways related to cardiometabolic syndrome. While we utilized information predicted from functional non-coding variants for our analysis, functional studies are required to elucidate the molecular mechanisms of these variants and explore their potential utility as genomic biomarkers for constitutional types.

## Data Availability

All data from this study are included in the main figures, supplementary figures, and Supplementary tables.

## Supplementary Materials

The following supporting information can be downloaded at: https://www.mdpi.com/article/10.3390/biomedicines12061311/s1, Figure S1. Overall workflow of the genome-wide association study (GWAS); Figure S2. Functional variants of constitutional types; Table S1. Korea Sasang Constitutional Diagnostic Questionnaire 15; Table S2. Clinical information on constitutional types; Table S3. Genome-wide association study (GWAS) summary data and cnnGWAS scores for constitutional types; Table S4. Instrumental variables (IVs) of exposures; Table S5. Single-nucleotide polymorphism (SNP) effects of outcomes; Table S6. Two sample MR results of constitutional types on stroke subtypes; Table S7. STROBE-MR checklist. References [67,68] are cited in the supplementary materials
