# Supplementary material for: Exploring Stroke Risk through Mendelian Randomization: A Comprehensive Study Integrating Genetics and Metabolic Traits in the Korean Population
Source: Biomedicines. 2024 Jun 13;12(6):1311. doi: 10.3390/biomedicines12061311 (PMC11201557; doi:10.3390/biomedicines12061311)
Supplement: Supplementary file 1 [file biomedicines-12-01311-s001.zip › legend_description.docx]

# Figure Legends

**Table S1. Korea Sasang Constitutional Diagnostic Questionnaire 15.** The KS-15 is a well-validated, shortened, and cost-effective screening instrument that is used to assess the constitutional type with clinical relevance (Cronbach a = 0.630). It consists of 15 items associated with individuals' anthropometric awareness of height and weight, 6 questions regarding personality (broad-minded/delicate, act quick/slow, active/passive, extraverted/introverted, masculine/feminine, excitable/rational), and 8 symptom-related questions concerning physiological functions (good digestion, appetite, excessive sweating, feeling when seated, abdominal tension during bowel movements, urination per night during sleep, dislike of cold and heat, preference for temperature when drinking water). The KS-15 derives the scores of each constitutional type, which range from 0 to 1.0 for each person; the sum of scores of the three constitutional types that is derived by the KS-15 for each individual is 1.0.

**Table S2. Clinical information on constitution types.** This table contains clinical information related to constitution types. The clinical characteristics of metabolic traits for each constitutional type are included.

**Table S3. Genome-wide association study (GWAS) summary data and cnnGWAS scores for constitution types**. This table contains a summary of data from GWAS on Constitution types.

**Table S4. Instrumental variables (IVs) of exposures.** This table shows the IVs for the constitutional type, which is the exposure used in the Mendelian randomization analysis. The most significant representative single nucleotide polymorphisms (SNPs) within the LD block, obtained after the clumping calculations, were selected based on the genome-wide association study (GWAS) results.

**Table S5. Single nucleotide polymorphism (SNP) effects of outcomes**. To assess the influence of instrumental variables (IVs) on outcomes, effect values were extracted from the genome-wide association study (GWAS) summary data related to Stroke risk factors used as the outcomes. In cases where a specific SNP was not present at the exact location, a proxy SNP with an *r^2^* value of ≥0.6 within the LD was selected. If the alternative allele types matched, the corresponding SNP information was extracted.

**Table S6.** A) **Two sample MR results of constitution types on stroke subtypes.** Different MR methods were used to evaluate the causal relationship between constitution types and stroke subtypes. MR methods include IVW (Inverse Variance Weighted), MR-Egger, Weighted median, Weighted mode. **B)** **Sensitivity analyses of constitution types on stroke subtypes**. To ensure that the association is not being disproportionately influenced by a single SNP, a leave-one-out sensitivity analysis has been conducted.

**Table S7. STROBE-MR checklist**. STROBE-MR (Strengthening the Reporting of Observational Studies in Epidemiology Using Mendelian Randomization) helps ensure clear and transparent reporting of MR studies. We presented the 20 items of the STROBE-MR checklist, their meaning and rationale, and attempted to clearly report the research procedures following the guide.

**Figure S1. Overall workflow of the genome-wide association study (GWAS). (a)** This figure illustrates the overall workflow of a GWAS. Single nucleotide polymorphism (SNP) datasets were constructed for the deep learning model. We selected the 30 most significant SNPs in the upstream and downstream regions (1 Mb distance) of each lead SNP (the most strongly associated SNP) from the GWAS results. Features not mapped to any SNP in more than 95% of the association blocks in each constitution type model were excluded. After organizing the dataset of significant SNPs, a feature was created to help understand how to use the functional area information provided by the cnnGWAS. The cnnGWAS model (a hierarchical pattern detector learning common pattern functions for disease-related locations) was used to detect functional patterns within the SNP block for each Constitution Type. The acquired functional scores were considered as the evidence of a closer association with the functional biological patterns within a constitution among the gene regions displaying statistical associations in the GWAS. (b) The performance of TE type, SE type, and SY type models measured based on AUC and F1 scores. The grey bars represent the original CNN model with only one convolutional layer, serving as lead SNPs. Model training and performance evaluation were conducted on training, validation, and test sets.

**Figure S2. Functional variants of constitution types.** (a) An outer-to-inner circle arrangement was made featuring the number of chromosomes and gene names, chromosome ideograms, functional variants for constitutional types (black: SE; red: TE; purple: SY), and diagrams of the overlapping portion of the constitution type. (b) The distribution of variants is shown as the functional annotation categories of Genetic Association Database (GAD).
