# Supplementary material for: Exploring Stroke Risk through Mendelian Randomization: A Comprehensive Study Integrating Genetics and Metabolic Traits in the Korean Population
Source: Biomedicines. 2024 Jun 13;12(6):1311. doi: 10.3390/biomedicines12061311 (PMC11201557; doi:10.3390/biomedicines12061311)
Supplement: Supplementary file 1 [file biomedicines-12-01311-s001.zip › Table S1.pdf]

Korea Sasang Constitutional Diagnostic Questionnaire 15; KS-15

A. *The following gathers your demographic information. Please fill in the blank or mark the appropriate number.*

**General Info**	Sex	1□ male 2□female	**Age**	( ) years
**Body Type**	Height	☐☐☐.☐ cm (Round to first decimal point)	Weight	☐☐☐.☐ kg (Round to first decimal point)
	**BMI**	☐☐☐.☐ kg/m² ※Weight(kg)÷{Hight(m) X Height(m)}		

B. *The following questions require that you reflect on your own personality in order to determine your Sasang constitution type. Answer each question by marking the number that best describes your personality. Mark 2 when you express each trait equally.*

[Example] Do you tend to give up easily or endure?	①give up easily	✔②**moderate**	③endure
2. Is your personality bold or delicate?	1☐bold	2☐moderate	3☐delicate
3. Do you tend to act quickly or slowly?	1☐quick	2☐moderate	3☐slow
4. Is your personality active or passive?	1☐active	2☐moderate	3☐passive
5. Is your personality outgoing or introverted?	1☐outgoing	2☐moderate	3☐introverted
6. Is your personality masculine or feminine?	1☐masculine	2☐moderate	3☐feminine
7. Are you rational or irrational when it comes to decision making?	1☐irrational	2☐moderate	3☐rational

C. *The following questions are for tendencies that have occurred over the* <u>*last 6 months*</u>*.You may ask the study staff for clarification if any of the questions are difficult to answer.*

8. How well is your digestion?

$_1$☐Very well $_2$☐Not very well, but it does not cause me significant discomfort $_3$☐Bad, and it causes me significant discomfort

9. How is your appetite?

$_1$☐Good $_2$☐ Moderate $_3$☐Not good

10. How much do you sweat in general?

$_1$☐A lot $_2$☐Average $_3$☐Not much

11. How do you feel after sweating?

$_1$☐Refreshed $_2$☐Tired $_3$☐No difference

12. How often is it hard to endure the abdominal tension before bowel movement?

$_1$☐Often $_2$☐Sometimes $_3$☐Never

13. How often do you wake up (during the night) to urinate?

$_1$☐Never $_2$☐Once $_3$☐More than twice

14. Which do you dislike more; the cold or the heat?

$_1$☐I dislike the cold $_2$☐I dislike the heat $_3$☐I dislike both; or I am fine with both

15. How do you prefer the temperature of your usual drinking water?

$_1$☐Warm $_2$☐Cold $_3$☐Doesn’t matter
