# Supplementary material for: Exploring Stroke Risk through Mendelian Randomization: A Comprehensive Study Integrating Genetics and Metabolic Traits in the Korean Population
Source: Biomedicines. 2024 Jun 13;12(6):1311. doi: 10.3390/biomedicines12061311 (PMC11201557; doi:10.3390/biomedicines12061311)
Supplement: Supplementary file 1 [file biomedicines-12-01311-s001.zip › Table S7.pdf]

Item No.	Section	Checklist item	Page No.	Relevant text from manuscript
1	**TITLE and ABSTRACT**	Indicate Mendelian randomization (MR) as the study's design in the title and/or the abstract if that is a main purpose of the study	1	Title: Exploring Stroke Risk Through Mendelian Randomization: A Comprehensive Study Integrating Genetics and Metabolic Traits in the Korean Population<br><br>Purpose: In this study, we compared the estimates obtained for different groups by applying the Mendelian random method; this was performed in order to investigate the causal effect that genetic characteristics have on stroke, according to the constitution type.
	**INTRODUCTION**			
2	**Background**	Explain the scientific background and rationale for the reported study. What is the exposure? Is a potential causal relationship between exposure and outcome plausible? Justify why MR is a helpful method to address the study question	1,2	The cardiometabolic condition is considered to be a potent modifiable risk factor, as the development of stroke in this case can be prevented; for example, the blood pressure can be lowered via weight loss in people with obesity and via reductions in the systolic and diastolic blood pressure [6-8].<br><br>Exposure: Constitution Type Group(Risk factor cardiometabolic condition), Outcome : Stroke
3	**Objectives**	State specific objectives clearly, including pre-specified causal hypotheses (if any). State that MR is a method that, under specific assumptions, intends to estimate causal effects	2	In this study, we aimed to investigate constitution types and their contribution to the risk of stroke, thereby enhancing the comprehension of stroke etiology and providing insights for the development of more effective preventive measures
	**METHODS**			
4	**Study design and data sources**	Present key elements of the study design early in the article. Consider including a table listing sources of data for all phases of the study. For each data source contributing to the analysis, describe the following:	3,6	Method Section 2.3, 2.4, 2.5, Figure 2

		a)	Setting: Describe the study design and the underlying population, if possible. Describe the setting, locations, and relevant dates, including periods of recruitment, exposure, follow-up, and data collection, when available.	2,3,	2.1. Study Participants<br>2.4. Genetic Instrument Variables<br>2.5. Outcomes
		b)	Participants: Give the eligibility criteria, and the sources and methods of selection of participants. Report the sample size, and whether any power or sample size calculations were carried out prior to the main analysis	2	2.1. Study Participants
		c)	Describe measurement, quality control and selection of genetic variants	3	2.4. Genetic Instrument Variables
		d)	For each exposure, outcome, and other relevant variables, describe methods of assessment and diagnostic criteria for diseases	3	2.5. Outcomes
		e)	Provide details of ethics committee approval and participant informed consent, if relevant	9	Institutional Review Board Statement:
5	**Assumptions**		Explicitly state the three core IV assumptions for the main analysis (relevance, independence and exclusion restriction) as well assumptions for any additional or sensitivity analysis	4	2.6. MR and Sensitivity Analyses
6	**Statistical methods: main analysis**		Describe statistical methods and statistics used	2,3,4	
		a)	Describe how quantitative variables were handled in the analyses (i.e., scale, units, model)	2	2.2. Clinical Data Analysis<br>2.6. MR and Sensitivity Analyses
		b)	Describe how genetic variants were handled in the analyses and, if applicable, how their weights were selected	3	2.4. Genetic Instrument Variables<br>2.5. Outcomes<br>Table S3, Table S4
		c)	Describe the MR estimator (e.g. two-stage least squares, Wald ratio) and related statistics. Detail the included covariates and, in case of two-sample MR, whether the same covariate set was used for adjustment in the two samples	3,4	2.6. MR and Sensitivity Analyses<br>Table S5
		d)	Explain how missing data were addressed	4	Linkage disequilibrium proxy SNPs were used when a specific SNP was not present in a dataset, with the criteria set as r2 > 0.6 and the minor allele frequency (MAF) > 0.01. To harmonize the exposure and resulting SNP effects, incorrect effect allele types were removed, and palindromic SNPs were considered.
		e)	If applicable, indicate how multiple testing was addressed	4	The MR Egger method enables the horizontal pleiotropy to be determined and presents a causal effect that is unbiased by imbalances or directional

				effects across all SNPs. Moreover, the median-based estimator ensures the unbiased estimation of the causal effect.
7	**Assessment of assumptions**	Describe any methods or prior knowledge used to assess the assumptions or justify their validity	4	2.6. MR and Sensitivity Analyses
8	**Sensitivity analyses and additional analyses**	Describe any sensitivity analyses or additional analyses performed (e.g. comparison of effect estimates from different approaches, independent replication, bias analytic techniques, validation of instruments, simulations)	4	The analysis was performed using the MRbase [47] application, which includes several sensitivity analyses to assess horizontal pleiotropy and other assumption violations.
9	**Software and pre-registration**			
	a)	Name statistical software and package(s), including version and settings used	4	2.6. MR and Sensitivity Analyses
	b)	State whether the study protocol and details were pre-registered (as well as when and where)	4	2.6. MR and Sensitivity Analyses
	**RESULTS**			
10	**Descriptive data**			
	a)	Report the numbers of individuals at each stage of included studies and reasons for exclusion. Consider use of a flow diagram	6	Figure 2
	b)	Report summary statistics for phenotypic exposure(s), outcome(s), and other relevant variables (e.g. means, SDs, proportions)	7	Figure 3, Table S3, Table S4
	c)	If the data sources include meta-analyses of previous studies, provide the assessments of heterogeneity across these studies	6,7	Figure 2, Figure 3
	d)	For two-sample MR:<br>i. Provide justification of the similarity of the genetic variant-exposure associations between the exposure and outcome samples<br>ii. Provide information on the number of individuals who overlap between the exposure and outcome studies	6	3.3. Causal Relationship Between Constitution Type and Stroke Obtained Through the MR Analysis<br>Table S3, Table S4
11	**Main results**			
	a)	Report the associations between genetic variant and exposure, and between genetic variant and outcome, preferably on an interpretable scale	7	Figure 3
	b)	Report MR estimates of the relationship between exposure and outcome, and the measures of uncertainty from the MR analysis, on an interpretable scale, such as odds ratio or relative risk per SD difference	7	Figure 3, Table S5

		c)	If relevant, consider translating estimates of relative risk into absolute risk for a meaningful time period	7	Figure 3, Table S5
		d)	Consider plots to visualize results (e.g. forest plot, scatterplot of associations between genetic variants and outcome versus between genetic variants and exposure)	7	Figure 3
12	**Assessment of assumptions**				
		a)	Report the assessment of the validity of the assumptions	6	Table S5
		b)	Report any additional statistics (e.g., assessments of heterogeneity across genetic variants, such as I^2, Q statistic or E-value)	6	Table S5
13	**Sensitivity analyses and additional analyses**				The analysis results were not included in the paper, but can be provided as a supplement table if necessary.
		a)	Report any sensitivity analyses to assess the robustness of the main results to violations of the assumptions		Table S5
		b)	Report results from other sensitivity analyses or additional analyses		Table S5
		c)	Report any assessment of direction of causal relationship (e.g., bidirectional MR)		Table S5
		d)	When relevant, report and compare with estimates from non-MR analyses		Table S5
		e)	Consider additional plots to visualize results (e.g., leave-one-out analyses)		Table S5
	**DISCUSSION**			8	
14	**Key results**		Summarize key results with reference to study objectives	8	4. Discussion
15	**Limitations**		Discuss limitations of the study, taking into account the validity of the IV assumptions, other sources of potential bias, and imprecision. Discuss both direction and magnitude of any potential bias and any efforts to address them	8	5. limitation
16	**Interpretation**				
		a)	Meaning: Give a cautious overall interpretation of results in the context of their limitations and in comparison with other studies	8	5. limitation
		b)	Mechanism: Discuss underlying biological mechanisms that could drive a potential causal relationship between the investigated exposure and the outcome, and whether the gene-environment equivalence assumption is reasonable. Use causal language carefully, clarifying that IV estimates may provide causal effects only under certain assumptions	8	5. limitation

		c) Clinical relevance: Discuss whether the results have clinical or public policy relevance, and to what extent they inform effect sizes of possible interventions	8	5. limitation
17	**Generalizability**	Discuss the generalizability of the study results (a) to other populations, (b) across other exposure periods/timings, and (c) across other levels of exposure	NA	
	**OTHER INFORMATION**			
18	**Funding**	Describe sources of funding and the role of funders in the present study and, if applicable, sources of funding for the databases and original study or studies on which the present study is based	9	
19	**Data and data sharing**	Provide the data used to perform all analyses or report where and how the data can be accessed, and reference these sources in the article. Provide the statistical code needed to reproduce the results in the article, or report whether the code is publicly accessible and if so, where	9	
20	**Conflicts of Interest**	All authors should declare all potential conflicts of interest	9	

This checklist is copyrighted by the Equator Network under the Creative Commons Attribution 3.0 Unported (CC BY 3.0) license.

1. Skrivankova VW, Richmond RC, Woolf BAR, Yarmolinsky J, Davies NM, Swanson SA, et al. Strengthening the Reporting of Observational Studies in Epidemiology using Mendelian Randomization (STROBE-MR) Statement. JAMA. 2021;under review.

2. Skrivankova VW, Richmond RC, Woolf BAR, Davies NM, Swanson SA, VanderWeele TJ, et al. Strengthening the Reporting of Observational Studies in Epidemiology using Mendelian Randomisation (STROBE-MR): Explanation and Elaboration. BMJ. 2021;375:n2233.
