# Supplementary figures and images for: Exploring Stroke Risk through Mendelian Randomization: A Comprehensive Study Integrating Genetics and Metabolic Traits in the Korean Population
Source: Biomedicines. 2024 Jun 13;12(6):1311. doi: 10.3390/biomedicines12061311 (PMC11201557; doi:10.3390/biomedicines12061311)

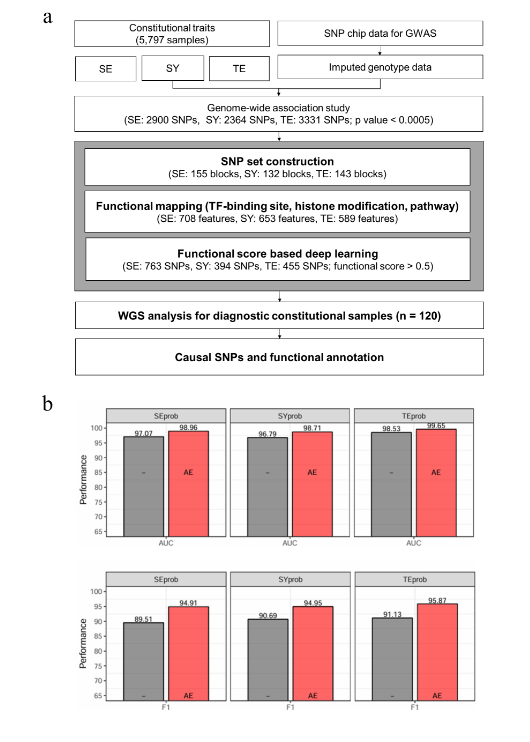

Supplement: Supplementary file 1 [file biomedicines-12-01311-s001.zip › Figure S1.tiff]

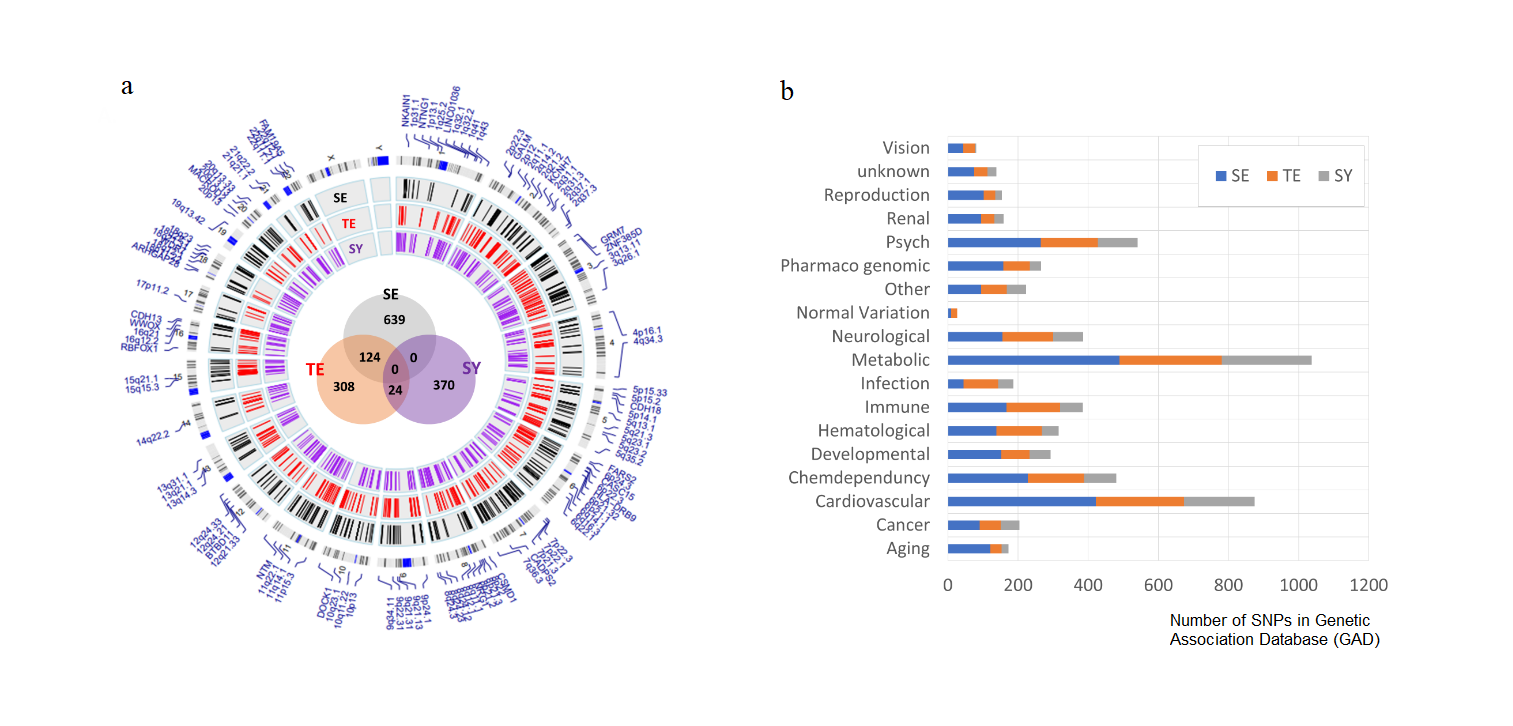

Supplement: Supplementary file 1 [file biomedicines-12-01311-s001.zip › Figure S2.tiff]
